# *Salmonella* isolated from individual reptiles and environmental samples from terraria in private households in Sweden

**DOI:** 10.1186/1751-0147-56-7

**Published:** 2014-01-24

**Authors:** Veronica O Wikström, Lise-Lotte Fernström, Lennart Melin, Sofia Boqvist

**Affiliations:** 1 Department of Biomedical Sciences and Veterinary Public Health, Swedish University of Agricultural Sciences, PO Box 7028, Uppsala, SE 750 07, Sweden; 2 Department for Bacteriology, National Veterinary Institute, Uppsala, SE 751 89, Sweden

**Keywords:** *Salmonella*, Reptile, Public health, Bacteriological culture, Environmental samples, Cloacal samples

## Abstract

**Background:**

This study investigates *Salmonella* spp. isolated from privately kept reptiles and from environmental samples such as bedding materials or water from the floor of the enclosures (terraria). It also compares isolation of *Salmonella* using Modified Semisolid Rappaport-Vassiliadis (MSRV) medium or selective enrichment in Rappaport-Vassiliadis-Soya (RVS) pepton broth. Cloacal swabs or swabs from the cloacal area were collected from 63 individual reptiles belonging to 14 households. All reptiles were from different terraria and from 62 of these, environmental samples were also collected. Sampling were done by the reptile owners according to written instructions and sent by mail immediately after sampling. All but three samples were analyzed within 24 h after collection. Colonies suspected for *Salmonella* were tested for agglutination and serotyped using the White-Kauffmann-Le Minor scheme. The relative sensitivity (*se*) and specificity (*sp*) for MSRV compared with RVS, and the agreement coefficient kappa (*κ*) were calculated.

**Results:**

*Salmonella* was isolated from 50/63 (80%) terraria, either from the reptiles (31/63; 49%) or from bedding material (39/62; 63%). The most common subspecies was *Salmonella enterica* subspecies *enterica* followed by *S. enterica* subspecies *diarizonae*. In reptiles, the most common *S. enterica* subspecies *enterica* serovars were Java (n = 4) and Fluntern (n = 4), compared with the serovars Tennessee (n = 10) and Fluntern (n = 10) in the environmental samples. The exact same set of *Salmonella* subspecies and serovars were not isolated from the individual reptiles and the environmental samples from any of the households. Isolation using MSRV yielded more *Salmonella* isolates 61/113 (54%) than enrichment in RVS 57/125 (46%). The *se* was 97.9% (95% Confidence Interval 93.9-100), the *sp* 78.5% (95% CI 68.5-88.5) and the *κ* 0.74, indicating substantial agreement between the tests.

**Conclusions:**

*Salmonella* can be expected to be present in environments where reptiles are kept. This constitutes public health risks and should be considered during handling of the reptiles and during cleaning and disposal of bedding. A combination of different culturing techniques may be used to increase the isolation rate.

## Background

Infection with *Salmonella* in reptiles has mainly been addressed as a public health hazard due to the zoonotic potential of the bacteria. The infection in reptiles is often subclinical although symptoms such as necrotizing enteritis and death of the animal may occur [1]. Reptiles carry *Salmonella* in their intestinal tract and may shed the bacteria intermittently [2,3]. The bacterium has also been isolated from, for example, cloacae, skin or water in terraria [2,4,5]. Humans can be infected directly through handling of the animal or indirectly through contaminated surroundings [6-8].

Six percent of all reported human salmonellosis cases in Sweden between 1998 and 2000 have been estimated to be associated with reptiles [9]. The same Population Attributable Fraction (PAF) has been reported from the US [7], whereas studies from the UK and the Netherlands have shown that less than one percent of reported human cases were attributed to reptiles [10,11]. Although these figures are relatively low, they do represent a large number of cases. Children below the age of five are of particular risk and it has been shown that the majority of reported human cases of Reptile Associated Salmonellosis (RAS) occur in this age group [9,10,12]. From the US it has been estimated that the PAF for reptile or amphibian contact was 11% for all sporadic salmonellosis cases in persons <21 years of age [7].

*Salmonella* is frequently isolated from reptiles kept in private homes and a study from Italy showed that 24% of reptiles at a sale centre carried *Salmonella* in their gastro-intestinal tract [13] and 63% of reptiles sampled in private homes or pet shops [5]. In Austria *Salmonella* was isolated from 54% of reptiles sampled in their home environment [4]. *Salmonella* can also be isolated from the surroundings in which the reptiles are kept [14]. It is not only handling of the reptiles but also contact with the terrarium/cage, for example during cleaning, that may constitute public health risks. With this in mind, there is limited data, to the authors’ knowledge, on presence of *Salmonella* in the terraria in private households.

To improve the readability of this paper all serovars belonging to *Salmonella enterica* subspecies (subsp.) *enterica* are denoted by the name given in the White-Kauffmann-Le Minor Scheme [15] e.g. *S.* Typhimurium. Subspecies belonging to *S. enterica* subsp. *arizonae, diarizonae, houtenae* and *salamae* are denoted *S. arizonae, S. diarizonae, S. houtenae and S. salamae* respectively, without their antigenic formula.

This study investigates prevalence of *Salmonella* in privately owned reptiles and in the terrarial environment in Sweden. It also compares the serovars isolated from individual reptiles and from environmental samples in the terraria, and investigates the importance of using different culturing media.

## Materials and methods

### Study design and data collection

Information about the study was published on five Swedish reptile associated websites and reptile owners willing to participate in the study were encouraged to contact the first author (VOW) through email or telephone. Reptile owners who agreed to participate in the study received detailed written sampling instructions, materials for sampling (small plastic container, plastic spoon, and Aimes agar gel medium transport swabs (Copan Italia S.P.A, Brescia, Italy) by mail one to two weeks before sampling. They also received a short written questionnaire to collect epidemiological information of the sampled reptiles and they were informed about the possibility of withdrawal from the study at any time. The study was conducted during December 2011 and sampling was done on Mondays to Wednesdays to ensure arrival of samples before weekends. From each household a maximum of ten reptile terraria were included and from each of these one reptile and one environmental sample was collected by the owner. The reptile samples consisted of swabs of the cloacae (n = 26), of the cloacal area (n = 36), or of swabs from recently delivered faeces (n = 2). The swabs were placed in Aimes medium immediately after sampling. All but three environmental samples consisted of bedding material. The remaining three were water collected from the floor of the enclosure. All samples were placed in plastic containers, equivalent to 25 g of sample materials, marked with date and time of sampling and sent by ordinary mail. All but three samples were analysed within 24 h after collection. The study was approved by the Ethics Committee in Uppsala, Sweden (Dnr C167/11) and the reptile owners approved to the study by collecting and sending the samples.

### Bacteriological analyses

The swabs with cloacal or faecal materials were diluted in 10 ml buffered pepton water (BPW; Oxoid, Hampshire, UK) and the environmental samples in 225 ml BPW. All samples were subjected to pre-enrichment at 37 ± 1°C for 18 ± 2 h. From here, two different culture methods were used. In the first method, 0.1 ml of BPW was diluted in 10 ml pre-heated Rappaport-Vassiliadis-Soya pepton broth (RVS; Oxoid, Hampshire, UK) and incubated at 41.5 ± 0.5°C for 24 ± 3 h. Two full loops (20 μl) of RVS was streaked on Xylose Lysine Deoxycholate agar with Novobiocin (XLD + N; Lab M, Lancashire, UK; Sigma Aldrich Co., Stockholm, Sweden) and Brilliant green (BG; Oxoid, Hampshire, UK) agar plates, respectively, and incubated at 37 ± 1°C for 24 ± 3 h. In the second method, 0.1 ml of BPW was dispersed in three equal sized drops on Modified Semisolid Rappaport-Vassiliadis medium (MSRV; Oxoid, Hampshire, UK; Sigma Aldrich Co., Stockholm, Sweden) agar plates and incubated at 41.5 ± 0.5°C for 24 ± 3 h. In case of negative culture the plates were re-incubated at 41.5°C for another 24 ± 3 h. One micro litre of colony materials from the opaque zone of the colonies was streaked on XLD + N and BG, and incubated at 37 ± 1°C for 24 ± 3 h.

Colonies suspected as *Salmonella* spp. on the XLD + N and/or BG plates were subcultured on Bromecresole Purple Lactose agar (Lab M, Lancashire, UK; Oxoid, Hampshire, UK; Merck, Darmstadt, Germany) plates and incubated at 37 ± 1°C for 24 ± 1 h. *Salmonella* spp. suspected colonies, one from the XLD + N plate and one from the BG plate, were tested for agglutination with polyvalent *Salmonella* O and H serum. The colonies confirmed as *Salmonella* spp. were serotyped according to the White-Kauffmann-Le Minor scheme. All analyses, apart from serotyping, were performed at the Department of Biomedical Sciences and Veterinary Public Health, Swedish University of Agricultural Sciences, Uppsala, Sweden. Serotyping was performed at the Department for Bacteriology at the National Veterinary Institute, Uppsala, Sweden.

### Data analysis

The RVS method (NMKL 71.5.1999) was used as the reference test against which the isolation rate obtained from the MSRV method (NMKL 187.2007) was compared. The relative sensitivity (*se*), the relative specificity (*sp*) and the level of agreement (kappa; κ) between the isolation methods were calculated using Win Episcope 2.0. An almost perfect test would give a κ >0.8 whereas a slight agreement would give a value <0.2 [16].

## Results

### Descriptive results

In total, 14 households participated in the study. Sixty three individual reptiles were sampled, one per terrarium, and 62 environmental samples were collected (Table 1). All but three households had more than one terrarium, each with several reptiles. In total, 25 snakes, 37 lizards and one turtle were included, representing 20 reptile species. Data on age and sex was known for 61 and 55 reptiles, respectively. Of those were 42 (69%) between one and six years of age, and 36 (65%) were females and 19 (35%) males. Eighty one percent of the reptiles were carnivores and the remaining were omnivores or herbivores. Data on the origin were known for 53 of the reptiles and of those were 32 (60%) from private breeders in Sweden, 15 (28%) were imported and 6 (11%) bought from pet shops.

**Table 1:** **Number of terraria and reptiles included in a study investigating the presence of *Salmonella *** **spp. in privately kept reptiles in Sweden**

**Household**	**n reptile species**	**n terraria**	**n reptiles**
		**(n samples)**	**(n samples)**
1	5	5 (5)	10 (5)
2	1	1 (1)	1 (1)
3	5	7 (7)	35 (7)
4	3	6 (6)	11 (6)
5	2	12 (9)	18 (9)
6	4	9 (3)	13 (3)
7	1	20 (10)	18 (10)
8	1	1 (1)	1 (1)
9	1	2 (1)	6 (1)
10	6	7 (3)	14 (3)
11	1	3 (3)	5 (3)
12	3	2 (3)	25 (3)
13	5	17 (9)	19 (9)
14	1	1 (1)	1 (1)

### *Salmonella* isolated from reptiles and the terrarium environment

In total, *Salmonella* was isolated from 11 (79%) of the households and from 50 (80%) of terraria (from the reptiles and/or the environmental samples). *Salmonella* was isolated from 24 (96%) out of 25 terraria with snakes and from 26 (70%) out of 37 with lizards. All isolated *Salmonella* belonged to *S. enterica* and the most common subspecies was *S. enterica* subsp. *enterica*. This was followed by *S. diarizonae* in terraria with snakes and *S. salamae* in terraria with lizards. From all but one household being positive for *Salmonella*, up to three subspecies of *S. enterica* and four serovars of *S. enterica* subsp. *enterica* were isolated (Table 2).

**Table 2:** ***Salmonella *** **spp. isolated from reptiles and terrarial environmental samples in private households**

	**Individual samples**		**Environmental samples**	
**Household**	**Subspecies**	***Enterica *** **serovar**	**Subspecies**	***Enterica *** **serovar**
	**(n isolates)**		**(n isolates)**	
1	*Diarizonae* (2)		-	
	*Enterica* (1)	Kisarawe	*Enterica* (1)	Kisarawe
	*Enterica* (1)	Java	*Enterica* (2)	Java
2	*Enterica* (1)	Kisaware	-	
3	*-*		*Diarizonae* (1)^h^	
	*Enterica* (1)^a^	Edinburg	-	
	*Enterica* (1)^a^	Fluntern	*Enterica* (2)^g, h^	Fluntern,
	-		*Enterica* (1)	Kentucky
	*Enterica* (2)	Muenchen	*Enterica* (3)	Muenchen
	*Doutenae* (1)		*Houtenae* (1)^g^	
4	*Diarizonae* (3)		*Diarizonae* (1)	
	-		*Enterica* (2)	Kentucky
	-		*Enterica* (1)	Tennesse
	*Salamae* (1)		*-*	
5	*Houtenae* (1)		-	
	*Enterica* (2)^b^	Fluntern	*Enterica* (3)	Fluntern
	*Salamae* (2)^b^		*Salamae* (2)	
6	*Diarizonae* (3)^c, d^		*Diarizonae* (1)	
	*Enterica* (1)^d^	Newport	*-*	
	*Enterica* (1)^c^	Java	-	
7	-		*Arizonae* (1)^i^	
	*Diarizonae* (3)^e^		*Diarizonae* (3)^i^	
	*Enterica* (1)	Java	*-*	
	-		*Enterica* (1)^j^	Pomona
	*Enterica* (1)^e^	Tennesse	*Enterica* (5)^j^	Tennesse
8	-		-	
9	*-*		*Enterica* (1)	Fluntern
10	*-*		*Arizonae* (2)^k^	
	*-*		*Diarizonae* (1)^k^	
11	-		*-*	
12	*Enterica* (1)^f^	Fluntern	*Enterica* (1)	Fluntern
	-		*Enterica* (1)	unknown
	*Houtenae* (1)^f^		*-*	
	*Salamae* (1)		*Salamae* (1)	
13	*Diarizonae* (2)		*Diarizonae* (1)	
	*Enterica* (1)	Java	*Enterica* (2)^m^	Java
	*Enterica* (1)	Muenchen	*Enterica* (1)	Muenchen
	-		*Enterica* (1)^m^	Tennesse
	-		*Enterica* (1)^l^	Victoria
	*Salamae* (1)		*Salamae* (2)^l^	
14	-		-	

^a-m^two subspecies isolated from one individual.

-not detected.

In total, *Salmonella* was isolated from 31 (49%) of the reptiles. From six individuals, two serovars of *S. enterica* subsp. *enterica* were isolated, thus 37 isolates were identified in total (Table 2). Among the reptiles the most common subspecies was *S. enterica* (n = 16) followed by *S. diarizonae* (n = 12), *S. salamae* (n = 5) and *S. houtenae* (n = 3). In total, seven serovars belonging to *S. enterica* subsp. *enterica* were isolated, the most common being *S*. Java (n = 4) and *S.* Fluntern (n = 4). The isolation rate was higher from cloacal samples (61%) as compared with swabs from the cloacal area (42%). The two faecal samples were *Salmonella* negative.

Thirty nine (63%) of the environmental samples were *Salmonella* positive, all being bedding materials. Two serovars of *S. enterica* subsp. *enterica* were isolated from seven households, thus 46 isolates from the bedding materials in the terraria were obtained (Table 2). The most common subspecies was *S. enterica* (n = 29), followed by *S. diarizonae* (n = 8), *S. salamae* (n = 6), *S. arizonae* (n = 3) and *S. houtenae* (n = 1). The most common *S. enterica* serovars were *S.* Tennesse (n = 10) and *S.* Fluntern (n = 10). Only in two households were *Salmonella* isolated only from the bedding materials and not from the reptiles.

The exact same set of *Salmonella* subspecies and serovars were not isolated from the reptiles and the bedding materials from any of the households (Table 2). However, the same *Salmonella* were isolated from the reptiles and the bedding materials in 17 (45%) out of 38 terraria.

### Comparison between MSRV and RVS agar

The *Salmonella* isolates obtained using MSRV media and selective enrichment in RVS are shown in Table 3. In total, 61/113 (54%) samples from reptiles and bedding materials were positive for *Salmonella* using MSRV. Using selective enrichment in RVS resulted in *Salmonella* being isolated from 57 (46%) out of 125 samples. Of the 113 samples from reptiles or bedding materials that were analysed using both MSRV and RVS, 62 (55%) were *Salmonella* positive. *Salmonella* was isolated from both MSRV and RVS in 47 cases (42%). The relative *se* for MSRV compared with RVS was 97.4% (95% Confidence Interval 93.9% - 100%) and the relative *sp* was 78.5% (95% CI 68.5% - 88.5%). The κ coefficient was 0.74 which indicates substantial agreement between the two isolation methods.

**Table 3:** ***Salmonella *** **spp. isolates from reptiles and the terrarial environment in private households using different culturing techniques**

***Salmonella *** **spp.**	**n positive reptile sample**		**n positive terrarium sample**		**n total positive**	
	**MSRV**^**1**^	**RVS**^**2**^	**MSRV**	**RVS**	**MSRV**	**RVS**
*S. arizonae*			2	1	2	1
*S. diarizonae.*	10	9	6	6	16	15
*S. enterica*						
Serovar Edinburg		1				1
Serovar Fluntern	3	2	7	4	10	6
Serovar Java	2	4	2	3	4	7
Serovar Kentucky			3	1	3	1
Serovar Kisarawe		2		1		3
Serovar Muenchen	3	2	3	3	6	5
Serovar Newport	1				1	
Serovar Pomona				1		1
Serovar Tennesse		1	6	4	6	5
Serovar Victoria			2		2	
Unknow serotype			1		1	
*S. houtenae*	2	2		1	2	3
*S. salamae*	5	4	3	5	8	9
**Total**	26	27	35	30	61	57

^1^Modified Semisolid Rappaport-Vassiliadis medium.

^2^Rappaport-Vassiliadis-Soya pepton broth.

## Discussion

This study showed that *Salmonella* was present in 80% of all households keeping reptiles as pets. *Salmonella* was isolated from both the reptiles and from the bedding materials in all but two terraria. These results indicate that *Salmonella* can be present in the majority of households keeping reptiles in Sweden even if the isolation rate from reptiles was found to be lower (49%). The isolation rate obtained in this study is similar to other studies in which *Salmonella* was isolated from 24 to 63% of sampled reptiles [4,5,17]. However, in these studies environmental samples were not investigated.

We found that *Salmonella* was isolated more often from the environmental samples compared with the reptile samples. One reason is likely that environmental samples reflect *Salmonella* carried and shed by all reptiles sharing the same terrarium. This emphasise the importance of keeping a good hygiene when disposing of faecal materials and water from reptile terraria or cages [18]. The importance of environmental *Salmonella* transmission was shown in a study reporting an outbreak of salmonellosis among children visiting an open reptile exhibition in which the majority of the reported cases had not been in direct contact with the reptiles [8]. Another study from a zoo confirmed that reptiles are important as spreaders of *Salmonella* in their surroundings [14]. Although an individual animal is culture negative it cannot be excluded that the particular animal constitute a health hazard as *Salmonella* can be shed intermittently [3]. Also, a negative test may not ensure safety due to possibilities for re-exposure from other infected reptiles in the terrarium or from an environment contaminated with *Salmonella*. Repeated sampling is thus required before concluding that an animal is free from infection. A wide range of serovars was isolated from the reptiles, which is in agreement with findings from other studies [4,5]. Results obtained also support the suggestion that cold-blooded animals generally are the main reservoirs for *S. salamae*, *S. diarizonae*, *S. arizonae* and *S. houtenae*[14]. The serovar most commonly isolated from reptiles and environmental samples in the present study was *S*. Fluntern. This serovar has occasionally been reported from reptiles [2,13]. A study from Denmark reported serovars well-known to be pathogenic for humans, such as *S*. Enteritidis, *S*. Typhimurium and *S*. Bovismorbificans [19] from reptiles. These serovars were not detected in the present study.

All reported domestic RAS cases in Sweden between 1990 and 2000 have been reviewed and found to be caused by 51 different *Salmonella* serovars [9]. *Salmonella* Enteritidis was the most frequent serovar accounting for 24% of all reported RAS cases, followed by *S*. Typhimurium (9%). None of these serovars were found in the present study. Of the serovars reported in that study [9] four were isolated in the present study as well, *S*. Newport (4.4% of all RAS cases), *S*. Muenchen (3.0% of all RAS cases), *S*. Java (2.7% of all RAS cases) and *S*. Pomona (0.3% of all RAS cases).

From the results obtained in this study it is obvious that keeping reptiles as pets may constitute public health risks. This is particularly true in households with young children as the risk of contracting *Salmonella* infection is higher for young children compared with adults [7,9,10]. Reasons why children are over represented are probably because of the handling and hygiene practises of young children [20]. It has also been shown that patients infected through turtles were younger than patients infected through snakes and lizards [9]. This probably reflects that certain types of reptiles, for example turtles, are more likely to be given to children as pets. It is recommended by the Centres for Disease Control and Prevention (CDC), Atlanta, USA that contact with reptiles should be avoided by children less than five years of age and that households with children in this age category shall not own reptiles even though transmission can be easily prevented through appropriate handling and hygiene [18].

Reptiles may carry different *Salmonella* serovars simultaneously [3,4], which also was shown in this study. Two serovars were isolated from several reptiles indicating that multi-serovar infection is common and two serovars were also isolated from the several samples of bedding material. It might well be that the number of isolated serovars were under diagnosed as only two colonies were tested from each sample of reptile or bedding material. The number of positive samples also depends on the culturing technique and it was found that use of MSRV yielded a higher number of isolates than selective enrichment in RVS. Other studies have also compared different culture media [14,17], however comparisons between studies are difficult as the selection of selective enrichments and culturing media differs among laboratories. The results may also be influenced by differences in media for culturing produced by different manufacturers. As it was found that the agreement between the two isolation methods were not perfect it is concluded that the highest isolation score will be obtained using a combination of different selective enrichment techniques and culturing media.

## Conclusions

*Salmonella* is often present in reptiles in Swedish household terraria. This constitutes public health risks and should be considered during handling of the reptiles or during cleaning and disposal of bedding. When culturing *Salmonella* a combination of different culturing techniques may be used to increase the isolation rate.

## Competing interests

The authors declare that they have no competing interests.

## Authors’ contributions

VOW participated in the design of the study, collected the materials, performed all laboratory analyses, interpreted the data and drafted the manuscript. LM helped with the laboratory analyses, assisted with summarising the results and commented on the manuscript. LLF participated in the design of the study, helped with the serotyping analyses and commented the manuscript. SB coordinated the study, participated in the design of it, helped in interpreting the results and drafted the manuscript. All authors read and approved the final manuscript.
